# Accuracy of tibial tuberosity-trochlear groove distance and tibial tuberosity-posterior cruciate ligament distance in terms of the severity of trochlear dysplasia

**DOI:** 10.1186/s13018-021-02527-x

**Published:** 2021-06-15

**Authors:** Conglei Dong, Chao Zhao, Ming Li, Chongyi Fan, Xunkai Feng, Kang Piao, Kuo Hao, Fei Wang

**Affiliations:** Department of Orthopaedic Surgery, Third Hospital of Hebei Medical University, Ziqiang Road 139, Shijiazhuang, 050051 China

**Keywords:** Patellar instability, Tibial tubercle to trochlear groove distance, Tibial tubercle to posterior cruciate ligament distance

## Abstract

**Purpose:**

Increased tibial tubercle-trochlear groove distance (TT-TG) was proposed as one of the main risk factors for patellofemoral instability (PFI). The increased TT-TG distance indicated externalization of the tibial tubercle with the reference of the trochlear groove. However, in the case of severe trochlear dysplasia, the reference point on the trochlear groove was indistinct, and the accuracy of TT-TG was controversial. The purpose of this study was to evaluate the accuracy of TT-TG and TT-PCL in consideration of the mild and severe trochlear dysplasia.

**Methods:**

From 2015 to 2020, MRI findings of consecutive knee joints with PFI symptoms diagnosed in our hospital were retrospectively analyzed. All knees with trochlear dysplasia were diagnosed by longitudinal MRI scan and lateral radiograph. The knees were classified according to the four-type classification system described by Dejour et al. Twenty cases of type A (mild trochlear dysplasia); 20 cases of type B, C, and D (severe trochlear dysplasia); and 20 cases of normal type were selected and divided into normal group (normal trochlea), mild group (type A), and severe group (type B, type C, type D). Tibial tubercle-trochlear groove distance (TT-TG), tibial tubercle-posterior cruciate ligament distance (TT-PCL), and the Dejour classification of trochlear dysplasia were assessed by 2 experienced orthopedics. The reliability of TT-TG distance and TT-PCL distance was tested by intraclass correlation coefficients (ICCs).

**Results:**

Comparing the differences between TT-TG and TT-PCL in the normal, mild, and severe groups, the TT-TG and TT-PCL in the mild and severe groups show different meanings (normal, 8.83 ± 3.62 mm vs. 8.44 ± 4.57 mm, *P* > 0.05; mild, 17.30 ± 4.81 mm vs. 20.09 ± 5.05 mm, *P* < 0.05; severe, 10.79 ± 4.24 mm vs. 12.31 ± 5.43 mm, *P* > 0.05). The Pearson correlation coefficient of TT-TG and TT-PCL measurements of trochlear dysplasia were r = 0.480 (mild group, *P* = 0.032) and r = 0.585 (severe group, *P* < 0.001). The intra-observer ICCs of TT-TG were r = 0.814 (mild group) and r = 0.739 (severe group). The inter-observer ICCs of TT-TG were r = 0.810 (mild group) and r = 0.713 (severe group). In the normal knee, the Pearson correlation coefficient of TT-TG and TT-PCL was r = 0.787(*P* < 0.001), the intra-observer ICC of TT-TG was r = 0.989, and the inter-observer ICC of TT-TG was r = 0.978.

**Conclusion:**

Compared with the mild trochlear dysplasia, the inter-observer and intra-observer correlations of TT-TG measurements decreased in the group of severe dysplastic trochlea (inter-observer ICC, 0.810 vs. 0.713; intra-observer ICC, 0.814 vs. 0.739). In the present study, the determination of TT-TG and TT-PCL distance are of great significance for patients with low-grade trochlear dysplasia. And TT-PCL, without referring to the abnormal trochlear groove, is an effective indicator to measure the lateralization of tibial tuberosity in patients with severe dysplastic trochlea.

## Introduction

High tibial tubercle-trochlear groove distance (TT-TG) is considered to be one of the main risk factors for patellofemoral dislocation [1]. In the case of trochlear dysplasia, TT-TG manifested as the externalization of the tibial tubercle and medialization of the trochlear sulcus, and it objectively described the dysregulation of the extensor mechanism [2]. TT-TG measurements are now used as decision support for adjusting surgery for patella dislocation [3]. Dejour et al. described a marked increase in TT-TG in patients with symptomatic patellar instability, characterized by patellar dislocation, which involves a complete loss of contact between the patella and the trochlear joint surface [4]. Using axial computed tomography (CT), the authors defined TT-TG as a pathological threshold of 20 mm (19.8 ± 1.6 mm), compared to a mean TT-TG measurement of 12.7 ± 3.4 mm in the control group. At the pathological threshold, 56% of the knee PFI showed a TT-TG value greater than 20 mm, compared to 3.4% in the control group. McNally et al. noted that patients with severe patella dislocation all had TT-TG above 20 mm [5]. Monk et al. recommended that the TT-TG threshold be set at 14.5 mm in patients with patella instability [6]. Some authors have found significant variation in TT-TG even in healthy patients with the normal shape of the trochlear groove. Some authors found that the normal value of TT-TG was 13.0 ± 5.0 mm, and the authors used CT imaging [7–9]. Wittstein et al. found a normal value of 9.4 ± 0.6 mm, and Pandit et al. described a normal value of 10 ± 1 mm [10, 11]. The latter author analyzed axial magnetic resonance imaging (MRI) scans and showed good internal and internal reliability. Historically, TT-TG was measured using axial CT scanning. At present, MRI is considered to be an equivalent imaging method [12]. Recently, Camp et al. have shown that it is possible to measure TT-TG in MRI and CT with good mutual reliability [13]. Besides, the authors described that the TT-TG values measured by MRI were lower than the TT-TG values measured by CT. In their work, Dejour et al. had pointed out that TT-TG measurements lacked precision [14]. They describe the lack of accuracy as a result of the difficulty in determining the nadir of the trochlear groove in patients with severe trochlear dysplasia. They describe the lack of accuracy as a result of the difficulty in determining the nadir of the trochlear groove in patients with severe trochlear dysplasia. These statements are supported by the impression made in clinical practice when the observer attempted to measure TT-TG in severe trochlear dysplasia. However, since TT-TG was used as decision support in clinical practice, the decreased accuracy would affect surgical decision-making and surgical outcome [15]. Tibial tubercle-posterior cruciate ligament distance (TT-PCL) is a measure of coronal plane misalignment and has been considered as an alternative to TT-TG because of concerns about the reliability of TT-TG in knee flexion and rotation. TT-PCL is the length of a line from the TT origin, parallel to the posterior condyle line of the tibia, to the most medial of the origin of PCL in the tibia [16]. Since TT-PCL refers to 2 points on the tibia, it is a measure of pure lateralization of the tibial tuberosity and is not affected by any limb rotation in the setting of knee motion. Therefore, we calculate the inter-observer and intra-observer correlation coefficients of TT-TG and TT-PCL measurements, and differences between groups, and hypothesize the use of TT-PCL as surgical decision support in patients with severe trochlear dysplasia.

## Materials and methods

**Fig. 1:**
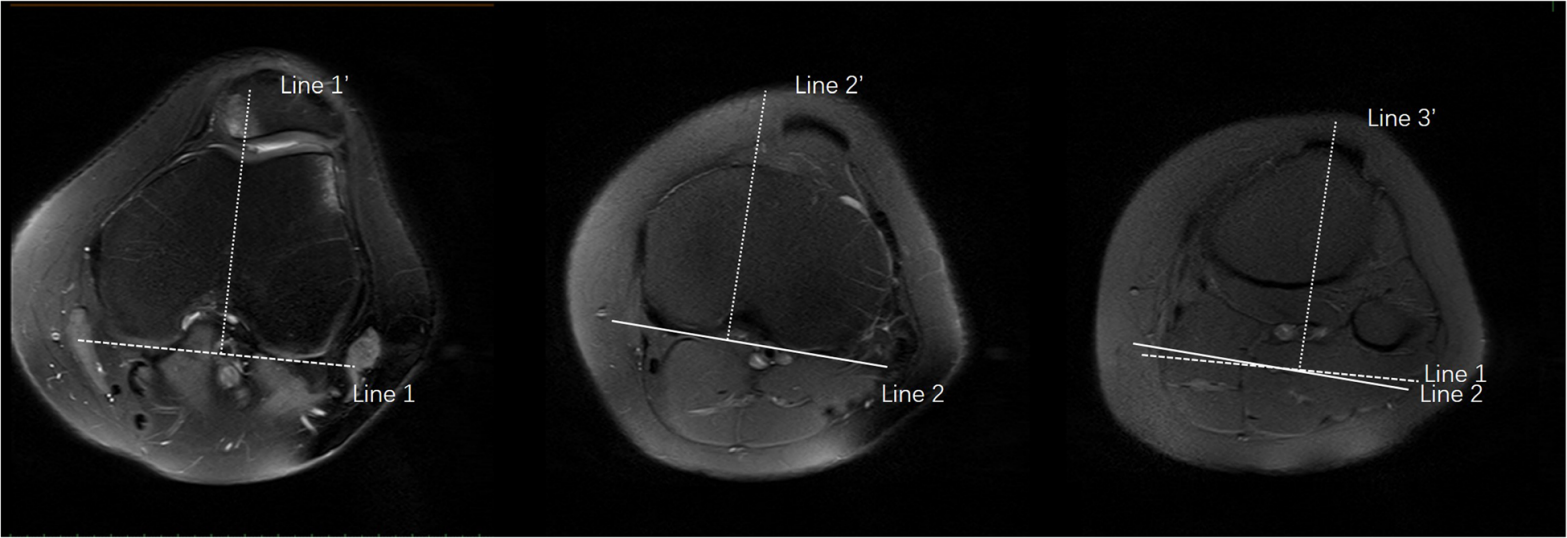
TT-TG and TT-PCL measurement methods. Line 1’ was drawn through the deepest point of the trochlear groove and perpendicular to the cortical edge of the bilateral posterior condyle (line 1). Line 2’ was drawn through the tibial insertion of the posterior cruciate ligament and perpendicular to the posterior edges of the proximal tibia. The distance between the line through the top of the tibial tubercle (line 3’) perpendicular to line 1 and line 1’ under the overlaid images was defined as tibial tuberosity-trochlear groove (TT-TG) distance. The distance between line 3’ perpendicular to line 2 and line 2’ under the overlaid images was defined as tibial tuberosity-posterior cruciate ligament (TT-PCL) distance

The protocol of the study was approved by the Academic Ethics Committee of the Third Hospital of Hebei Medical University. Informed written consent was acquired from all individuals prior to participating in the study. Sixty consecutive MRI scans were retrospectively analyzed, including 40 patients with patellofemoral dislocation and 20 patients without patellofemoral dislocation. Prior to MRI, no patients in the patellofemoral dislocation group underwent distal reduction or osteotomy. The PFI manifestations of 40 patients with trochlear dysplasia of the knee were evaluated by longitudinal MRI scan and true lateral radiograph. Magnetic resonance imaging (MRI) with the patient in a supine position with 1.5 T units (VA17ASymphonyATIM System, Simeon, Germany) was performed. During the MRI scan, the knee was fully extended. The scans were performed in a fat-saturated proton density-weighted fast spin-echo imaging sequence under standardized conditions. To assess trochlear dysplasia, an axial MRI scan was performed at the distal femoral transition where the full width of the trochlear cartilage was visible. Measurements were made using GE Central PACS-IW (General Electric Company, Connecticut). The software allows measurement accuracy to one decimal place. The knees are distributed according to the four-type classification system described by Dejour [17]. TT-TG and TT- PCL measurements were performed in all 60 knees. All scans were read by two observers (observers 1 and 2). Two observers were orthopedic surgeons with surgical experience in the treatment of patellofemoral dislocation. The observer is blind to the results of the other observer (inter-observer correlation). Observer 1 double reads two different occasions (inter-observer correlation). Finally, inter-observer and intra-observer correlation coefficients of TT-TG and TT-PCL measurements and differences between groups were calculated. Radiological measurements for patellofemoral disorders and patellar shapes were as follows [18, 19]. First, the location of the farthest MRI with the visible insertion of the patellar tendon into the tibial tubercle was selected for tibial tubercle measurement. The insertion of the patellar tendon into the center serves as a reference point for measuring the TT portion. To define the deepest point within the trochlear groove, the trochlear groove (TG) measurements were taken from an axial MRI at the proximal end, with a complete cartilaginous trochlear (Fig. 1). The posterior condyle line of the femur was established on this image, and the line perpendicular to it was drawn through the center of the lowest point of the trochlear groove, while the line perpendicular to the tangent line of the posterior condyle was drawn by TT. The distance between the two perpendicular lines, namely the distance between the patellar tendon and the cartilage trochlear groove, which is measured in millimeters, is called TT-TG.

In the TT-PCL measurement, the common sources of tibial nodules were the same as those described above. For the posterior cruciate ligament (PCL) measurement, an axial MRI was performed at the farthest end of the tibia with a clear image of PCL origin, and then the posterior condyle line of the bone tibia was mapped on the axial MRI, just distal to the articular surface. The vertical line originates from this line through the innermost PCL (Fig. 1). TT-PCL is the length of a line from the common TT origin, parallel to the posterior condyle line of the tibia, to the most medial of the origin of PCL in the tibia.

**Fig. 2:**
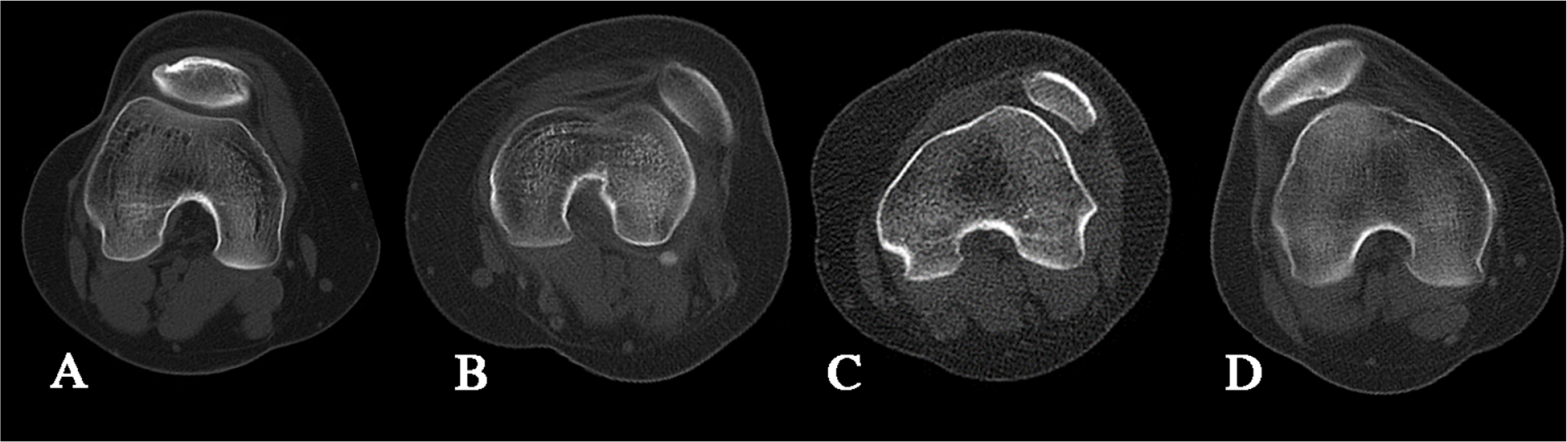
The Dejour classification. **A** CT showed the Dejour classification of trochlear dysplasia, type A. **B** CT showed the Dejour classification of trochlear dysplasia, type B. **C** CT showed the Dejour classification of trochlear dysplasia, type C. **D** CT showed the Dejour classification of trochlear dysplasia, type D

Using axial and sagittal MRI in patients with each block form of identification and classification of Dejour, the following was found: the normal femoral trochlear (normal), superficial femoral trochlear (type A, Fig. 2A), flat femoral trochlear joint with a trochlear spur (type B, Fig. 2B), a dominant and convex lateral femoral trochlear facet (type C, Fig. 2C), or complete lack of medial trochlear facet joints with spurs on the femoral trochlear (type D, Fig. 2D). In identifying the structure of the lateral and medial pulleys on axial MRI, images of the proximal end of the intact cartilaginous pulley were used. There were 20 cases of type A; 20 cases of type B, C, and D (severe trochlear dysplasia); and 20 cases of normal type that have been calculated and measured, and these were divided into normal group (normal), mild group (type A), and severe group (type B, type C, type D).

The knee flexion angle in the scanner did not affect the TT-TG measurement. The presence or absence of knee effusion was a potential confounding of other measurement variables, but it was not found to alter our measurement results.

## Statistical analysis

Inter-observer and intra-observer correlation coefficients (ICCs) are used to calculate the Kappa statistics of the statistical analysis measures for continuous measures and the discrete measures (such as Dejour classification). The simple linear regression model was used to evaluate the correlation. Between TT-PCL and TT-TG, all patients were grouped by trochlear morphology. The mean values of TT-TG and TTPCL were used to evaluate the severity of trochlear dysplasia by linear trend analysis. The differences in TT-TG and TT-PCL indexes between the PF group and the normal group were compared by an independent sample t test. All analyses and P values were calculated using SPSS version 22.0 (IBM, Armonk, NY). P values below 0.05 were considered statistically significant.

## Results

**Fig. 3:**
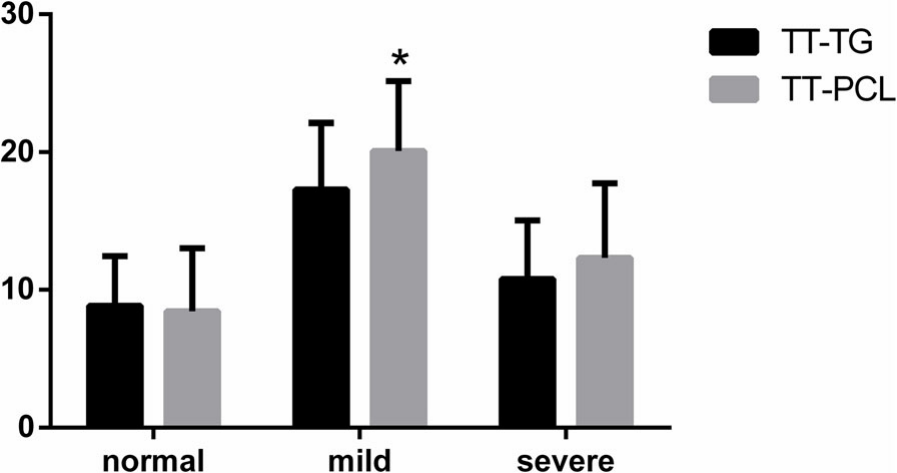
The comparison of TT-TG, TT-PCL, and Dejour classification. Asterisk means *p* < 0.05

There were 14 male and 6 female patients in the normal group, aged between 26 and 11 years (17.15 ± 4.21). The A type group included 6 male and 14 female patients, with an age range of 41 and 12 years (20.35 ± 6.84). The B, C, and D type group included 5 male and 15 female patients, with an age range of 31 and 13 years (19.10 ± 5.04). According to the Dejour classification system, the distribution of 60 knee joints is as follows: normal = 20, A type = 20, and B, C, and D type = 20. Observer 1 calculated the TT-TG mean ± the standard deviation values, 8.83 ± 3.62 mm for normal group, 17.30 ± 4.81 mm for the mild group, and 10.79 ± 4.24 mm for the severe group (severe vs. normal, *P* = 0.051; severe vs. mild, P < 0.001; normal vs. mild, *P* < 0.001). Observer 2 calculated the TT-PCL mean ± the standard deviation values, 8.44 ± 4.57 mm for normal group, 20.09 ± 5.05 mm for the mild group, and 12.31 ± 5.43 mm for the severe group (severe vs. normal, *P* < 0.001; severe vs. mild, *P* < 0.001; normal vs. mild, *P* < 0.001). Table 1 shows the morphology and group membership between the distribution groups of the femoral trochlea. Comparing the differences between TT-TG and TT-PCL in the normal, mild, and severe groups, the TT-TG and TT-PCL in the mild and severe group show different meanings (normal, 8.83 ± 3.62 mm vs. 8.44 ± 4.57 mm, *P* > 0.05; mild, 17.30 ± 4.81 mm vs. 20.09 ± 5.05 mm, *P* < 0.05; severe, 10.79 ± 4.24 mm vs. 12.31 ± 5.43 mm, *P* > 0.05). Figure 3 shows the comparison of TT-TG, TT-PCL, and Dejour classification. The Pearson correlation coefficient of TT-TG and TT-PCL measurements of trochlear dysplasia were r = 0.480 (mild group, *P* = 0.032) and r = 0.585 (severe group, *P* < 0.001). The intra-observer ICCs of TT-TG were r = 0.814(mild group) and r = 0.739(severe group). The inter-observer ICCs of TT-TG were r = 0.810(mild group) and r = 0.713(severe group). In the normal knee, the Pearson correlation coefficient of TT-TG and TT-PCL was r = 0.787(*P* < 0.001), the intra-observer ICC of TT-TG was r = 0.989, and the inter-observer ICC of TT-TG was r = 0.978.

**Table 1:** The morphology and group membership between the distribution groups of the femoral trochlea

Trochlear dysplasia classification	Knee	Gender (men/women)	Age (years)
Normal group	20	14/6	17.15 ± 4.21
Mild group	20	6/14	20.35 ± 6.84
Severe group	20	5/15	19.10 ± 5.04

## Discussion

In clinical practice, TT-TG measurements are often used to make surgical decisions about soft tissue stabilization and distal rearrangement [20]. This finding suggests that in patients with severe trochlear dysplasia, it seems the results obtained by TT-TG and TT-PCL are inconsistent, which will affect clinical judgment. In the present study, the intra-observer and inter-observer ICC of TT-TG measurements in patients with type B, C, and D trochlear dysplasia suggest that TT-TG measurements are not accurate in patients with severe trochlear dysplasia. Enlarged TT-TG is considered to be one of the major risk factors for patellofemoral dislocation, so its measurement should be effective [21]. In 1994, Dejour et al. found a significant increase in TT-TG in patients with patellofemoral joint instability [14]. In Dejour’s work, patellar instability was defined as a complete loss of contact between the trochlear groove and the patellar articular surface. The authors described a cut-off value of approximately 20 mm (19.8 ± 1.6 mm) for TT-TG. Until now, TT-TG > 20 mm has still been considered pathological. Besides, the authors reported that the mean TT-TG of asymptomatic subjects in the control group was 12.7 ± 3.4 mm, which was made on superimposed axial CT [2]. At the same time, MRI has replaced CT as the most advanced technology [22]. MRI includes more advanced imaging techniques that, in addition to measuring TT-TG, can also assess concomitant soft tissue injury and articular cartilage condition [23]. This may help make preoperative surgical decisions, in the preoperative stages. Schoettle et al. noted that CT or MRI can be used for TT-TG measurements, and they reported good internal, periodic, and inter-methodological reliability of TT-TG measurements in a comparative evaluation of CT and MRI. Camp et al. calculated the inter-observer ICC of TT-TG measurements in CT and MRI and found TT-TG values are more reliable in MRI than in CT scan [24, 25]. For TT-TG measurements, the intra-observer ICC decreased significantly with the increase in the severity of trochlear dysplasia.

To make accurate measurements of TT-TG, the trochlear groove must be definable in addition to locating the cranial portion of the tibial tubercle and its attachment to the patellar tendon on a transverse MRI scan [26]. For TT-TG measurements, the deepest point of the trochlear groove must be identified on the first coronal MRI transverse slice depicting a complete layer of cartilage, especially in flat trochlear (such as type B) or even in protruding trochlear (types C and D), and the definition of the deepest point in a pulley groove is very variable. The imprecision of the positioning of the trochlea groove is reflected in the decreased intra-observer ICC in higher levels of trochlea dysplasia. We detected a decrease in the inter-observer ICC of TT-TG measurements as the severity of trochlear dysplasia increased. In severe trochlear dysplasia, the value of inter-observer ICC appears to be even lower than the intra-observer ICC. This difference may be due to the increasing inconsistency of observer definitions of trochlear grooves in severe trochlear dysplasia. In addition to this, another issue must be considered, especially in terms of the TT-TG interpretation: an increase in the severity of trochlear dysplasia is accompanied by an increase in the asymmetry of the trochlear surface. In the asymptomatic knee, the first transverse MRI coronal section showed almost symmetry of the medial and lateral trochlear facets. While in the dysplastic trochlear, the lateral trochlear facets showed a much longer time than the medial facet [27]. The asymmetry of the trochlear surface increases with the increase of the severity of trochlear dysplasia. Nelitz et al. demonstrated the correlation between the Dejour classification system and the facet joint asymmetry parameters [28]. This asymmetry of the control lateral facet implies the innermost part of the trochlear groove and affects the measurement of true TT-TG. With the increase of the asymmetry of the trochlear surface, the inner transfer of the trochlear groove is more and more, leading to the high TT-TG. With this in mind, the high value of TT-TG in the case of trochlear dysplasia should not be used as decision support for tibial tubercle osteotomy. In patients with severe trochlear dysplasia, mainly proximal to the extensor mechanism, trochleoplasty should be considered. By deepening the trochlear, the symmetry of the facet can be restored, resulting in the lateralization of the trochlear groove, and reducing the maladjustment and normalization of TT-TG. In routine clinical practice, knee trochlear should be classified, and TT-TG values become ineffective in patients with severe trochlear dysplasia classified by anterolateral and axial MRI radiographs. In patients with trochlear dysplasia, the lesion cannot be resolved by tibial nodule osteotomy alone.

There are several limitations worth mentioning in this study. Our control group was not completely asymptomatic. MRI scans of these patients were due to meniscus tears or cartilage damage. Patients with the patellofemoral chief complaint or anterior knee pain were excluded. Furthermore, the observer could not turn a blind eye to whether the MRI scan was obtained in patients in the control group or the PFI group since the morphological findings were evident in the latter case. Another limiting conclusion is that TT-TG detection is of great significance for mild trochlear dysplasia. TT-TG measurements are increasingly inaccurate in severe trochlear dysplasia, especially preoperatively. Therefore, the final decision to perform tibial nodular osteotomy should consider Dejour classification and TT-PCL measurement as an alternative to TT-TG. The TT-PCL is a method of quantifying tibial nodulation lateralization while avoiding the potential confounding effects of femoral rotation or knee flexion, which is often criticized by TT-TG. This study confirms the findings of previous studies that distinguish the “normal” (normal), “mild” (A), and “severe” (B, C, D) will increase the significance. However, this paper has no evidence to prove the advantages and disadvantages of the relationship between TT-TG and TT-PCL. It is also a pity that we did not come up with a better solution. We just found out in our clinical work that in the coronal assessment of patellofemoral instability, tibial tubercle-trochlear groove distance (TT-TG) and tibial tubercle-posterior cruciate ligament distance (TT-PCL) show different meanings when the femoral trochlea is stunted.

In conclusion, TT-TG is of great significance for mild trochlear dysplasia, but it has become increasingly inaccurate in the measurement of severe trochlear dysplasia, especially preoperatively. In severe trochlear dysplasia, TT-PCL can more accurately measure the position of tibial nodules because it is not affected by trochlear dysplasia. TT-TG was not statistically significant in severe trochlear dysplasia, while TT-PCL was statistically significant in severe trochlear dysplasia. In patients with severe trochlear dysplasia, TT-PCL has more clinical significance in the decision-making of tibial nodular osteotomy. Therefore, TT-PCL may challenge the use of TT-TG in some situations, especially in the case of severe femoral trochlear dysplasia. Further research is needed to confirm whether TT-TG is less sensitive and specific than TT-PCL for severe trochlear dysplasia.

## Data Availability

All of the data and materials are available.

## Abbreviations

KOA: Knee osteoarthritis
TT-TG: Tibial tubercle-trochlear groove distance
ICC: Intraclass correlation coefficients
CT: Computed tomography
TT-PCL: Tibial tubercle-posterior cruciate ligament distance
MRI: Magnetic resonance imaging

## References

[1] Machado SAF, Pinto RAP, Antunes A, de Oliveira PAR (2017). Patellofemoral instability in skeletally immature patients. Porto Biomed J.

[2] Wolfe S, Varacallo M, Thomas JD, Carroll JJ, Kahwaji CI (2021). Patellar instability. In: StatPearls. StatPearls Publishing Copyright© 2021.

[3] Nakamura S, Shima K, Kuriyama S, Nishitani K, Ito H, Matsuda S (2019). Tibial tubercle-trochlear groove distance influences patellar tilt after total knee arthroplasty. J Arthroplast.

[4] Caton JH, Dejour D (2010). Tibial tubercle osteotomy in patello-femoral instability and in patellar height abnormality. Int Orthop.

[5] McNally EG, Ostlere SJ, Pal C, Phillips A, Reid H, Dodd C (2000). Assessment of patellar maltracking using combined static and dynamic MRI. Eur Radiol.

[6] Monk AP, Doll HA, Gibbons CL, Ostlere S, Beard DJ, Gill HS, Murray DW (2011). The patho-anatomy of patellofemoral subluxation. J Bone Joint Surg Br.

[7] Hochreiter B, Hess S, Moser L, Hirschmann MT, Amsler F, Behrend H (2020). Healthy knees have a highly variable patellofemoral alignment: a systematic review. Knee Surg Sports Traumatol Arthrosc.

[8] Xu Z, Zhang H, Yan W, Qiu M, Zhang J, Zhou A (2021). Validating the role of tibial tubercle-posterior cruciate ligament distance and tibial tubercle-trochlear groove distance measured by magnetic resonance imaging in patients with patellar dislocation: a diagnostic study. Arthroscopy.

[9] Hernigou J, Chahidi E, Bouaboula M, Moest E, Callewier A, Kyriakydis T, Koulalis D, Bath O (2018). Knee size chart nomogram for evaluation of tibial tuberosity-trochlear groove distance in knees with or without history of patellofemoral instability. Int Orthop.

[10] Wittstein JR, O'Brien SD, Vinson EN, Garrett WE (2009). MRI evaluation of anterior knee pain: predicting response to nonoperative treatment. Skelet Radiol.

[11] Pandit S, Frampton C, Stoddart J, Lynskey T (2011). Magnetic resonance imaging assessment of tibial tuberosity-trochlear groove distance: normal values for males and females. Int Orthop.

[12] Thakkar RS, Del Grande F, Wadhwa V, Chalian M, Andreisek G, Carrino JA, Eng J, Chhabra A (2016). Patellar instability: CT and MRI measurements and their correlation with internal derangement findings. Knee Surg Sports Traumatol Arthrosc.

[13] Camp CL, Stuart MJ, Krych AJ, Levy BA, Bond JR, Collins MS, Dahm DL (2013). CT and MRI measurements of tibial tubercle-trochlear groove distances are not equivalent in patients with patellar instability. Am J Sports Med.

[14] Dejour H, Walch G, Nove-Josserand L, Guier C (1994). Factors of patellar instability: an anatomic radiographic study. Knee Surg Sports Traumatol Arthrosc.

[15] Hochreiter B, Hirschmann MT, Amsler F, Behrend H (2019). Highly variable tibial tubercle-trochlear groove distance (TT-TG) in osteoarthritic knees should be considered when performing TKA. Knee Surg Sports Traumatol Arthrosc.

[16] Seitlinger G, Scheurecker G, Högler R, Labey L, Innocenti B, Hofmann S (2012). Tibial tubercle-posterior cruciate ligament distance: a new measurement to define the position of the tibial tubercle in patients with patellar dislocation. Am J Sports Med.

[17] Van Haver A, De Roo K, De Beule M, Labey L, De Baets P, Dejour D, Claessens T, Verdonk P (2015). The effect of trochlear dysplasia on patellofemoral biomechanics: a cadaveric study with simulated trochlear deformities. Am J Sports Med.

[18] Beaconsfield T, Pintore E, Maffulli N, Petri GJ (1994). Radiological measurements in patellofemoral disorders. A review. Clin Orthop Relat Res.

[19] Panni AS, Cerciello S, Maffulli N, Di Cesare M, Servien E, Neyret P (2011). Patellar shape can be a predisposing factor in patellar instability. Knee Surg Sports Traumatol Arthrosc.

[20] Colatruglio M, Flanigan DC, Harangody S, Duerr RA, Kaeding CC, Magnussen RA (2020). Identifying patients with patella alta and/or severe trochlear dysplasia through the presence of patellar apprehension in higher degrees of flexion. Orthop J Sports Med.

[21] Shakespeare D, Fick D (2005). Patellar instability-can the TT-TG distance be measured clinically?. Knee.

[22] Hinckel BB, Gobbi RG, Filho EN, Pécora JR, Camanho GL, Rodrigues MB, Demange MK (2015). Are the osseous and tendinous-cartilaginous tibial tuberosity-trochlear groove distances the same on CT and MRI?. Skelet Radiol.

[23] Tan SHS, Lim BY, Chng KSJ, Doshi C, Wong FKL, Lim AKS, Hui JH (2020). The difference between computed tomography and magnetic resonance imaging measurements of tibial tubercle-trochlear groove distance for patients with or without patellofemoral instability: a systematic review and meta-analysis. J Knee Surg.

[24] Schoettle PB, Zanetti M, Seifert B, Pfirrmann CW, Fucentese SF, Romero J (2006). The tibial tuberosity-trochlear groove distance; a comparative study between CT and MRI scanning. Knee.

[25] Camp CL, Heidenreich MJ, Dahm DL, Bond JR, Collins MS, Krych AJ (2016). A simple method of measuring tibial tubercle to trochlear groove distance on MRI: description of a novel and reliable technique. Knee Surg Sports Traumatol Arthrosc.

[26] Dornacher D, Reichel H, Lippacher S (2014). Measurement of tibial tuberosity-trochlear groove distance: evaluation of inter- and intraobserver correlation dependent on the severity of trochlear dysplasia. Knee Surg Sports Traumatol Arthrosc.

[27] Keshmiri A, Schöttle P, Peter C (2020). Trochlear dysplasia relates to medial femoral condyle hypoplasia: an MRI-based study. Arch Orthop Trauma Surg.

[28] Nelitz M, Lippacher S, Reichel H, Dornacher D (2014). Evaluation of trochlear dysplasia using MRI: correlation between the classification system of Dejour and objective parameters of trochlear dysplasia. Knee Surg Sports Traumatol Arthrosc.

